# Rest and Disease

**Published:** 1893-01

**Authors:** 


### REST AND DISEASE.

"How do you feel after that bad illness of yours?" "Better than I have done for years ; it cleared it out of me." Some such conversation takes place between acquaintances every day. It might be difficult for either to say what the "it" was that the illness in question cleared out ; but the general meaning is clear—that the ex-invalid is better after his illness than he was before. Formerly he felt fatigue, lassitude, headache, dizziness ; now he is free from all these symptoms, he is fresh, active, buoyant, and he gives all the credit to the illness—the fever or pleurisy, the rheumatism or ague, which confined him to his bed for weeks. To say how organic disease can make any one more healthy would certainly pass the wit of man, but if one must explain phenomena most people will prefer to take the next preceding circumstance as the cause rather than go back to a further incident which carries with it an undeniable reproach. For, in truth, nature has been saying to our ex-invalid friend, "You are using yourself up too fast; I can't keep pace with you. I cannot supply tissue, blood, brain, as fast as you use them; do stop and let me make up on you." The man has answered—in deed if not in word—"I can't stop; I want to marry, and have not a sufficient income; I want to set up a carriage; I want to provide for my children; I love the excitement of work and money-making, and I won't stop." Then it comes to duel between man and nature, and nature wins. Man has only his will—a powerful weapon enough, but not omnipotent; nature has the support of every muscle, nerve, and cell in the body. Nature, Dame Nature, conquers, as women do, by sheer inertia, by refusing to comply with the impossible requirements of masculine will, by sitting down and weeping. Nature's tears are—disease.

In the old legend, Death gave the farmer three warnings of his coming—deafness, blindness and lameness. The farmer had not understood him, but that was his own fault; Death refused to grant him a longer delay because of his stupidity. Disease acts in much the same way as his sterner brother, with this difference, that if we take heed of its warnings, we may escape a more prolonged visitation. When the monitions of fatigue, headache, dyspepsia, sleeplessness, or whatever form of warning Nature may send are heeded—when sufficient rest, suitable food, pure air, and an absence of excitement are granted to the starved and overworked organism that craves for them, disease in its acuter forms passes by the door; when these warnings are neglected, it comes in and compels the rest the patient would fain refuse to take. You may "work off" a headache for the time, but you cannot work off a fever; you must lie down, and let it take its course. When after six weeks or so you return to work, feeling as if a burden had been lifted you, you give the fever the credit. "It cleared 'it' out of me," you declare—whatever the mystic "it" may be. Say rather the fever forced you to rest, and gave nature time to put "it"—arrears of energy—into you. Disease is not in itself a good, but it may be an opportunity for good to be done. This is the lesson of disease; happy are they who profit by it! Few of us, unfortunately, are wise enough to take the milder warning.—*Hospital.*

